# Prevalence, patterns, perceptions, and sociodemographic determinants of dietary supplement use among adults in the United Arab Emirates

**DOI:** 10.3389/fnut.2026.1955988

**Published:** 2026-10-13

**Authors:** Ali Alkhayat, Nelofar Sami Khan

**Affiliations:** College of Medicine, Gulf Medical University, Ajman, United Arab Emirates

**Keywords:** cross-sectional study, determinants, dietary supplements, health behavior, nutritional supplements, perceptions, prevalence, United Arab Emirates

## Abstract

**Background:**

Previous studies have documented dietary supplement use in the United Arab Emirates (UAE), but many have focused on specific populations, supplement categories, geographical settings, or particular contexts. Evidence integrating overall supplement use, patterns and experiences of use, perceptions, and associated sociodemographic and health-related factors among adult UAE residents remains limited. This study aimed to determine the prevalence and patterns of dietary supplement use, assess supplement-related perceptions, and identify factors associated with supplement use and perceptions among adults residing in the UAE.

**Methods:**

A cross-sectional, questionnaire-based study was conducted among 600 adults aged ≥18 years residing in the UAE (58.8% male, 41.2% female). Participants were recruited using convenience sampling through an anonymous electronic survey distributed through university-linked networks, community groups, and public online platforms. A structured questionnaire assessed sociodemographic and health characteristics, dietary supplement use and related practices, and four perceptions regarding supplement necessity, safety, disease prevention, and promotion by healthcare professionals. Associations were examined using Pearson’s chi-square test and binary logistic regression. Adjusted odds ratios (AORs) with 95% confidence intervals (CIs) were reported.

**Results:**

Overall, 287 participants (47.8%) reported ever using dietary supplements; among them, 58.9% were current users. Vitamins (70.6%), minerals (57.8%), and protein supplements (47.4%) were the most commonly reported categories. Managing nutritional deficiencies (38.0%) and improving fitness (35.3%) were the leading reasons for use, while healthcare professionals were the most frequently reported source of recommendation (40.1%). In the adjusted analysis, undergraduate/postgraduate education (AOR 18.32, 95% CI 9.09–36.89; *p* < 0.001), female sex (AOR 3.13, 95% CI 1.76–5.58; *p* < 0.001), Expat Arab nationality compared with UAE nationality (AOR 4.58, 95% CI 2.14–9.82; *p* < 0.001), and chronic medical conditions (AOR 2.15, 95% CI 1.27–3.64; *p* = 0.004) were associated with higher odds of supplement use, whereas employment compared with student status was associated with lower odds (AOR 0.28, 95% CI 0.13–0.61; *p* = 0.001). Ever supplement use was the only factor consistently associated with all four favorable perception outcomes (all *p* < 0.001).

**Conclusion:**

The findings highlight the need for evidence-based nutrition education and individualized professional guidance to support informed and appropriate use of dietary supplements. Incorporating supplement-use history into clinical assessment may help address inappropriate use, misconceptions, and potential supplement–drug interactions. Longitudinal and population-representative studies with documented geographic coverage across the UAE are needed to confirm these associations and clarify the relationship between supplement use and perceptions.

## 1 Introduction

Dietary supplements are products intended to supplement the usual diet by providing concentrated sources of nutrients or other substances with nutritional or physiological effects. They include vitamins, minerals, amino acids, fatty acids, probiotics, and herbal or botanical preparations, and are available in forms such as tablets, capsules, powders, and liquids (1, 2).

Dietary supplements are widely used across different populations, and their contribution to total nutrient intake makes their inclusion important in dietary assessment and nutrition monitoring (3). They are used for varied and sometimes overlapping reasons, including maintaining health, addressing perceived dietary inadequacies, supporting physical performance, and taking a more active role in health management (4). Interest also extends to healthy aging, although evidence of benefit varies considerably across supplements and health outcomes (5).

Dietary supplements can help prevent or correct nutrient deficiencies when supported by clinical evidence or public health recommendations, particularly among groups with specific nutritional requirements (1, 6). However, use outside evidence-based indications may provide little additional benefit while increasing the risk of excessive nutrient intake, supplement–drug interactions, adverse effects, unnecessary costs, and unrealistic expectations regarding disease prevention or treatment (6, 7). These concerns are particularly relevant because supplements are readily available through pharmacies, retail outlets, and online marketplaces, often without professional consultation (7, 8).

Supplement use is influenced by sociodemographic, lifestyle, health-related, and perceptual factors, although associations vary across populations because of differences in cultural practices, dietary habits, healthcare systems, regulatory environments, and access to supplements (9–12). Beliefs regarding effectiveness, safety, necessity, and appropriate use are also important. A recent systematic review identified perceived health benefits, confidence in product safety and efficacy, trust in information sources, and a desire for greater personal control over health as influences on supplement-use decisions (4). Supplement users have also been reported to hold more favorable perceptions of these products than non-users (13, 14).

Studies from Gulf Cooperation Council countries indicate substantial dietary supplement use, although prevalence and patterns vary according to population and study design (12, 15). In Kuwait, AlTarrah et al. (12) reported supplement use among 68.2% of young adults aged 18–35 years. Recent Saudi Arabian studies have further documented supplement use in specific populations and settings (16, 17).

In the UAE, Abdulla et al. reported that 37.8% of Dubai residents had ever used health supplements, and that use was associated with several sociodemographic and health-related factors (18). Other UAE studies have focused on college students (19), self-prescribed vitamin D supplementation among university students (20), and knowledge, perceptions, and use of protein supplements among university students (21). Radwan et al. (22) reported supplement use among 56.6% of 2,060 UAE adults during the COVID-19 pandemic, although that study specifically examined products used for COVID-19 prevention or treatment.

Despite this growing literature, available UAE studies differ considerably in geographical coverage, populations studied, supplement categories, and study context. Recent evidence integrating overall supplement use, multiple product categories, reasons and patterns of use, recommendation sources, user experiences, perceptions, and independent sociodemographic and health-related correlates in a single sample of adult UAE residents remains limited. An integrated assessment may therefore provide a more comprehensive understanding of supplement-use behaviors and inform locally relevant clinical counseling, nutrition education, and public health planning.

Therefore, this study aimed to determine the prevalence and patterns of dietary supplement use, assess perceptions regarding dietary supplements, and identify sociodemographic and health-related factors associated with supplement use and supplement-related perceptions among adults residing in the UAE. The study addressed the following research question: What are the patterns and perceptions of dietary supplement use among adults residing in the UAE, and which sociodemographic and health-related characteristics are associated with supplement use and supplement-related perceptions?

## 2 Methods

### 2.1 Study design, setting and participants

A cross-sectional, questionnaire-based study was conducted between June and October 2024 among adults aged ≥18 years residing in the UAE. Adults of both sexes and all nationalities residing in the UAE during the study period were eligible to participate. Individuals aged <18 years, those not residing in the UAE, and those who did not provide electronic informed consent were excluded.

A non-probability convenience sampling approach was used. The self-administered electronic questionnaire was initially disseminated through university-linked networks, including students who shared the survey link with family members, friends, and their wider social networks. The link was additionally circulated through community groups and public online platforms to broaden recruitment beyond the university population.

Although recruitment was intended to reach adults residing across the UAE through multiple electronic and community-based channels, the emirate of residence was not collected in the questionnaire. Consequently, the distribution of participants across the seven emirates could not be quantified, and geographic representativeness could not be assessed. The convenience and online recruitment strategy may also have favored individuals with greater digital access or interest in health-related topics, which should be considered when interpreting the generalizability of the findings.

The study was conducted in accordance with the principles of the Declaration of Helsinki. Ethical approval was obtained from the Institutional Review Board of Gulf Medical University, Ajman (Approval No. IRB-COM-STD-141-MAY-2024; 13 May 2024) before data collection. Electronic informed consent was obtained from all participants before accessing the questionnaire. Participation was voluntary and anonymous; participants could discontinue the survey at any time without consequence, and no personally identifiable information was collected. Study data were kept confidential, accessible only to the research team, and reported in aggregate form.

### 2.2 Sample size determination

The minimum required sample size was estimated using the formula for estimating a single population proportion (Kish formula). The calculation was based on a previously reported prevalence of dietary supplement use of 37.8% among adults in Dubai, a 95% confidence level, a relative precision of 10% of the estimated prevalence, and an anticipated non-response rate of 10%. Based on these assumptions, the required sample size was estimated to be 724 participants (18).

### 2.3 Questionnaire and data collection

Data were collected using a structured, self-administered electronic questionnaire developed with reference to previously published studies on dietary supplement use, consumer perceptions, and related health behaviors (12, 18, 19, 23). Items from these sources were adapted, where relevant, to the study objectives and the UAE context, while additional or modified questions were included to capture the supplement-use behaviors and perceptions examined in the present study. The adapted domains included sociodemographic and health characteristics, as well as questions on supplement use, including duration and frequency, product types and forms, reasons for use and discontinuation, sources of recommendation, and adverse effects. The complete questionnaire is provided as Supplementary Material.

The draft questionnaire was reviewed by four subject experts, two specialists in Community Medicine from Gulf Medical University and two general practitioners from Thumbay University Hospital, who assessed its content relevance, clarity, wording, and suitability for the target population. Their feedback was incorporated before pilot testing. A pilot study was subsequently conducted among 10 adults from the target population to assess comprehensibility, readability, clarity, and approximate completion time. Pilot participants were excluded from the final study sample, and minor modifications were made before the questionnaire was finalized. No internal-consistency coefficient was calculated because the perception items were analyzed as separate binary statements rather than combined into a multi-item scale.

The final questionnaire covered four major domains: (1) sociodemographic characteristics, including age, sex, marital status, nationality, education, occupation, monthly income, and health insurance; (2) health and lifestyle characteristics, including smoking, allergies, healthcare utilization, chronic medical conditions, and prescription medication use; (3) dietary supplement use and related practices, including awareness, current and previous use, duration and frequency, supplement categories, including vitamins, minerals, protein supplements, dietetic foods, energy drinks, sports nutrition products, herbal supplements, and other supplements, dosage forms, expenditure, reasons for use and discontinuation, sources of recommendation, perceived effectiveness, adverse effects, and regret following use; and (4) perceptions regarding dietary supplements, assessed using individual Agree/Disagree statements concerning their necessity, safety, disease-preventive role, and promotion by healthcare personnel. The questionnaire required approximately 10 min to complete.

The electronic survey link was distributed through the recruitment channels described above. Participation was voluntary and anonymous, and the survey platform was configured to permit one response per participant.

### 2.4 Statistical analysis

Data were entered into Microsoft Excel, checked for completeness and consistency, and analyzed using the Statistical Package for the Social Sciences (SPSS), version 29 (IBM Corp., Armonk, NY, United States). Categorical variables were summarized using frequencies and percentages. For descriptive analyses, dietary supplement use was categorized as current use, past use, or never used. For inferential analyses, the primary outcome was dietary supplement use, dichotomized as ever use (current and past users combined) versus never use, with never users serving as the reference category.

Associations between participant characteristics and dietary supplement use were initially examined using Pearson’s chi-square test. Binary logistic regression was used to identify factors associated with supplement use. Crude odds ratios (CORs) were obtained from univariable models. Covariates for the multivariable model were prespecified based on previous literature and their potential sociodemographic or health-related relevance to dietary supplement use, rather than selected solely on the basis of univariable statistical significance. The multivariable model included age, sex, nationality, occupation, health-insurance status, monthly income, educational level, frequency of healthcare visits, chronic medical conditions, prescription medication use, and smoking status. Adjusted odds ratios (AORs), 95% confidence intervals (CIs), and corresponding *p*-values were reported.

Separate binary logistic regression analyses were performed for each of the four dietary supplement perception statements, with Agree versus Disagree as the binary outcome. These assessed perceptions regarding whether dietary supplements are necessary for all ages, are generally harmless, help prevent chronic diseases, and should be promoted by healthcare professionals. Each adjusted perception model included the same 11 sociodemographic and health-related variables as the primary multivariable model, along with dietary supplement use status (ever versus never). The perception analyses were considered exploratory; therefore, no formal adjustment for multiple comparisons was applied, and the findings were interpreted cautiously.

Multicollinearity among the independent variables was assessed using tolerance and variance inflation factor (VIF) statistics. No evidence of problematic multicollinearity was identified (tolerance range: 0.449–0.943; VIF range: 1.060–2.228). Model performance for dietary supplement use was assessed using the Hosmer–Lemeshow goodness-of-fit test, Nagelkerke’s pseudo-*R*^2^, and overall classification accuracy. Adjusted associations with dietary supplement use were additionally presented as a forest plot of AORs with corresponding 95% CIs.

Analyses were conducted using available data for the variables included in each analysis; missing responses were not imputed. All statistical tests were two-tailed, and a *p*-value <0.05 was considered statistically significant.

## 3 Results

### 3.1 Prevalence of dietary supplement use

Among the 600 participants, 287 (47.8%) reported ever using dietary supplements, while 313 (52.2%) had never used them. Among ever users, 169 (58.9%) were current users and 118 (41.1%) were past users.

### 3.2 Sociodemographic and health characteristics

Table 1 summarizes the sociodemographic and health characteristics of participants according to dietary supplement-use status. Ever use differed according to sex, nationality, health-insurance status, monthly income, educational level, history of allergies, frequency of healthcare visits, chronic medical conditions, prescription medication use, smoking status, and knowledge of dietary supplements (all *p* < 0.05). No significant differences were observed according to age group, marital status, or occupation.

**Table 1:** Sociodemographic and health characteristics of participants according to dietary supplement-use status (*N* = 600).

Characteristic	Total *n* (%)	Never users *n* (%)	Ever users *n* (%)	*p*-value*	Current users *n* (%)	Past users *n* (%)	*p*-value^
Age group (*N* = 595)				0.656			0.268
Young adults (18–29 years)	372 (62.5)	197 (63.1)	175 (61.8)		98 (59.0)	77 (65.8)	
Middle-aged adults (30–49 years)	185 (31.1)	93 (29.8)	92 (32.5)		60 (36.1)	32 (27.4)	
Older adults (≥50 years)	38 (6.4)	22 (7.1)	16 (5.7)		8 (4.8)	8 (6.8)	
Sex (*N* = 600)				<0.001			0.002
Male	353 (58.8)	219 (70.0)	134 (46.7)		92 (54.4)	42 (35.6)	
Female	247 (41.2)	94 (30.0)	153 (53.3)		77 (45.6)	76 (64.4)	
Marital status (*N* = 599)				0.757			0.019
Single	367 (61.3)	193 (61.9)	174 (60.6)		112 (66.3)	62 (52.5)	
Married	232 (38.7)	119 (38.1)	113 (39.4)		57 (33.7)	56 (47.5)	
Nationality^†^ (*N* = 571)				<0.001			0.270
UAE national	331 (58.0)	173 (56.7)	158 (59.4)		97 (63.0)	61 (54.5)	
South Asian	150 (26.3)	116 (38.0)	34 (12.8)		16 (10.4)	18 (16.1)	
Expat Arab	90 (15.8)	16 (5.2)	74 (27.8)		41 (26.6)	33 (29.5)	
Occupation (*N* = 599)				0.124			0.285
Student	251 (41.9)	138 (44.2)	113 (39.4)		66 (39.1)	47 (39.8)	
Employed	329 (54.9)	168 (53.8)	161 (56.1)		98 (58.0)	63 (53.4)	
Currently not employed	19 (3.2)	6 (1.9)	13 (4.5)		5 (3.0)	8 (6.8)	
Health insurance (*N* = 589)				<0.001			0.065
No	114 (19.4)	80 (26.0)	34 (12.1)		15 (9.1)	19 (16.4)	
Yes	475 (80.6)	228 (74.0)	247 (87.9)		150 (90.9)	97 (83.6)	
Monthly income (*N* = 589)				<0.001			0.491
<AED 5,000	386 (65.5)	237 (76.2)	149 (53.6)		89 (54.6)	60 (52.2)	
AED 5,000–9,999	74 (12.6)	29 (9.3)	45 (16.2)		26 (16.0)	19 (16.5)	
AED 10,000–19,999	47 (8.0)	15 (4.8)	32 (11.5)		15 (9.2)	17 (14.8)	
≥AED 20,000	82 (13.9)	30 (9.6)	52 (18.7)		33 (20.2)	19 (16.5)	
Education (*N* = 598)				<0.001			0.805
High school or less	294 (49.2)	228 (73.3)	66 (23.0)		38 (22.5)	28 (23.7)	
Undergraduate/Postgraduate	304 (50.8)	83 (26.7)	221 (77.0)		131 (77.5)	90 (76.3)	
History of allergies (N = 600)				0.030			0.017
No	482 (80.3)	262 (83.7)	220 (76.7)		138 (81.7)	82 (69.5)	
Yes	118 (19.7)	51 (16.3)	67 (23.3)		31 (18.3)	36 (30.5)	
Doctor visits (*N* = 597)				<0.001			0.167
Once monthly	26 (4.4)	3 (1.0)	23 (8.1)		11 (6.5)	12 (10.3)	
Once every 2–6 months	188 (31.5)	83 (26.6)	105 (36.8)		57 (33.9)	48 (41.0)	
Once yearly or less	383 (64.2)	226 (72.4)	157 (55.1)		100 (59.5)	57 (48.7)	
Chronic medical condition (*N* = 600)				<0.001			<0.001
No	377 (62.8)	229 (73.2)	148 (51.6)		101 (59.8)	47 (39.8)	
Yes	223 (37.2)	84 (26.8)	139 (48.4)		68 (40.2)	71 (60.2)	
Prescription drug use during past month (*N* = 599)				0.005			0.591
No	498 (83.1)	273 (87.2)	225 (78.7)		134 (79.8)	91 (77.1)	
Yes	101 (16.9)	40 (12.8)	61 (21.3)		34 (20.2)	27 (22.9)	
Smoking status (*N* = 598)				<0.001			0.006
Non-smoker	258 (43.1)	100 (32.1)	158 (55.2)		81 (48.2)	77 (65.3)	
Current smoker	277 (46.3)	176 (56.4)	101 (35.3)		72 (42.9)	29 (24.6)	
Past smoker	63 (10.5)	36 (11.5)	27 (9.4)		15 (8.9)	12 (10.2)	
Knowledge of health supplements (*N* = 598)				<0.001			0.165
No	63 (10.5)	59 (19.0)	4 (1.4)		1 (0.6)	3 (2.5)	
Yes	535 (89.5)	252 (81.0)	283 (98.6)		168 (99.4)	115 (97.5)	

*Comparison between never and ever users.

^Comparison between current and past users.

Values are presented as *n* (%). Percentages represent column percentages. Pearson’s chi-square test was used for all comparisons. Variable-specific sample sizes (*N*) are shown because of occasional missing data.

^†^Nationality: Participants classified as Other/Mixed nationality (*n* = 29) were excluded from nationality-specific analyses because of the small sample size, allowing more stable estimates across the three predefined nationality groups.

Current vs. Past users: Comparisons between current and past users were restricted to participants who had ever used dietary supplements. Therefore, sample sizes differ from the overall never-versus-ever comparison.

A *p*-value <0.05 was considered statistically significant.

Among ever users, current and past users differed according to sex, marital status, history of allergies, chronic medical conditions, and smoking status (all *p* < 0.05). No significant differences were observed for the remaining characteristics.

### 3.3 Patterns of dietary supplement use

Among participants who had ever used dietary supplements, vitamins (70.6%) were the most commonly reported supplement category, followed by minerals (57.8%) and protein supplements (47.4%). Other supplement categories were reported less frequently (Figure 1).

**Figure 1:**
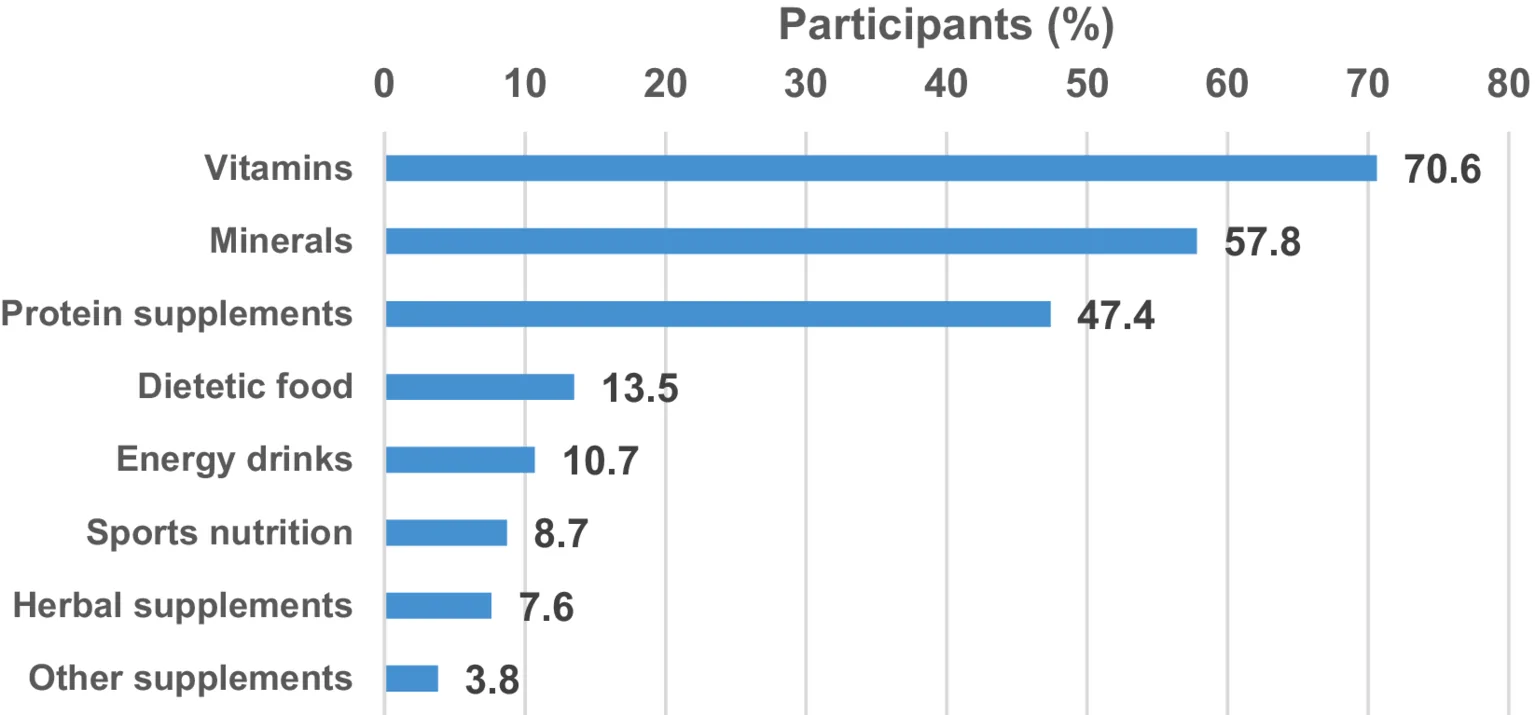
Categories of dietary supplements used among ever users (*n* = 287).

The most frequently reported reasons for dietary supplement use were managing nutritional deficiencies (38.0%) and improving fitness (35.3%), followed by pregnancy (10.7%). Doctors were the most frequently reported source of recommendation (40.1%), followed by friends (21.8%) and self-initiation (14.4%) (Figure 2).

**Figure 2:**
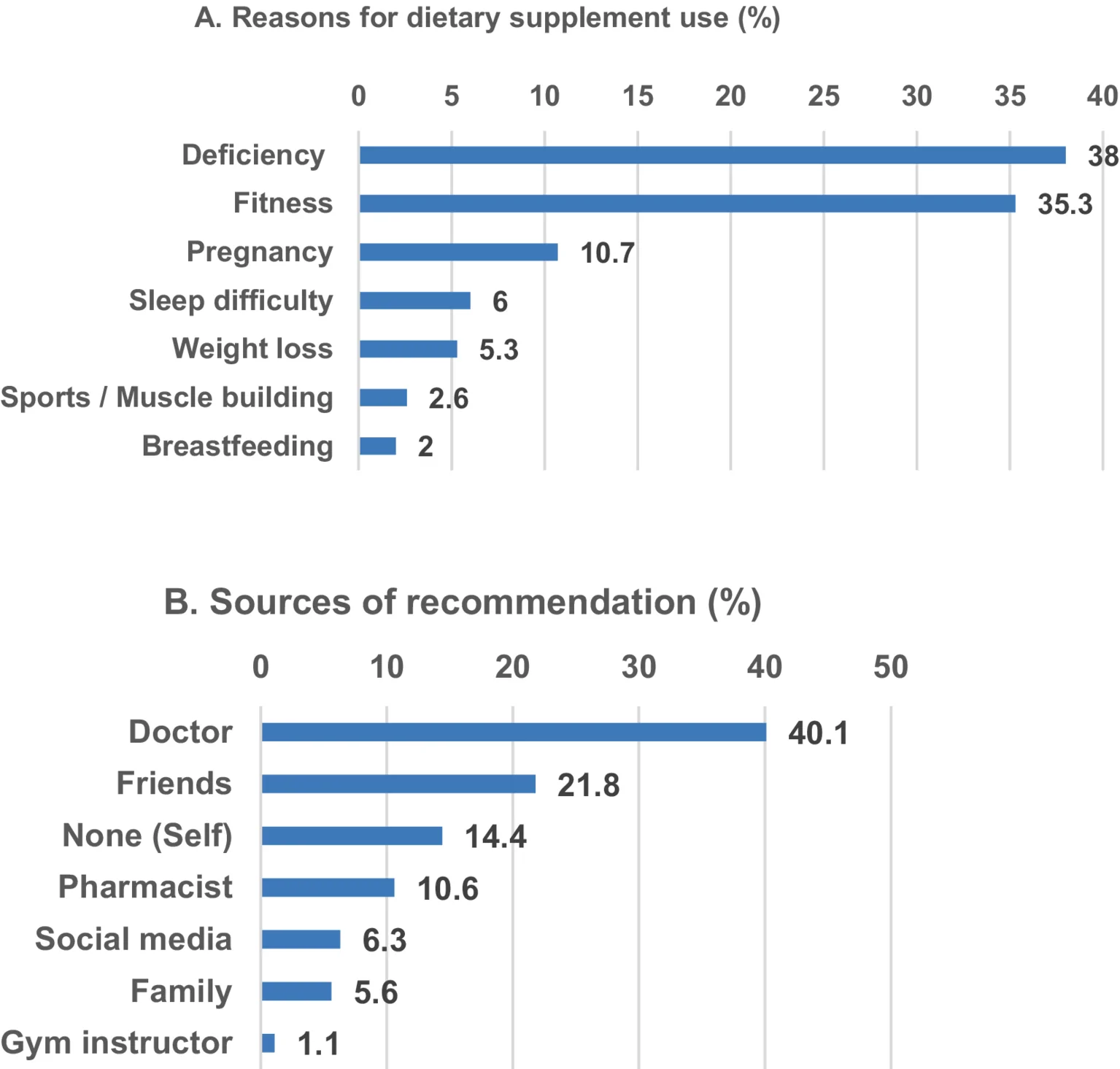
Reasons for dietary supplement use **(A)** and sources of recommendation **(B)** among ever users.

### 3.4 Sociodemographic correlates of specific dietary supplement categories

Table 2 summarizes the significant sociodemographic and health-related correlates of specific dietary supplement categories, with detailed analyses presented in Supplementary Tables S1A–H. Vitamin and mineral use was more frequent among women and married participants, whereas protein and performance supplement use was more frequent among men. Mineral and protein supplement use also varied significantly by age, while vitamin D use was more frequent among young adults. Folic acid use was associated with marital status, and iron use with sex, marital status, nationality, and health-insurance status. Calcium use was associated with nationality, occupation, health-insurance status, and chronic medical conditions. Income and smoking status were also associated with selected supplement categories. Educational level was not significantly associated with any of the individual supplement categories examined.

**Table 2:** Summary of significant sociodemographic correlates across different categories of dietary supplements.

Dietary Supplement category	Age	Sex	Marital status	Nationality	Occupation	Health insurance	Monthly income	Education*	Chronic disease	Smoking
Vitamins (overall)	–	Female ↑ (<0.001)	Married ↑ (0.001)	–	–	–	Significant association (<0.001)	–	–	Non-smoker ↑ (0.002)
Minerals (overall)	Middle-aged & older adults ↑ (0.020)	Female ↑ (<0.001)	Married ↑ (<0.001)	UAE Nationals ↑ (0.012)	Employed ↑ (0.024)	Insurance ↑ (0.005)	Significant association (<0.001)	–	–	Non-smoker ↑ (0.035)
Protein supplements	Middle-aged adults ↑ (0.017)	Male ↑ (<0.001)	–	–	Employed ↑ (0.003)	–	AED 5,000–9,999 ↑ (0.026)	–	–	Current smoker ↑ (<0.001)
Performance supplements (energy drinks + sports nutrition)	–	Male ↑ (<0.001)	Single ↑ (0.028)	–	–	–	AED 5,000–9,999 ↑ (0.033)	–	–	–
Vitamin D	Young adults ↑ (0.018)	–	–	–	–	No insurance ↑ (0.017)	–	–	–	–
Folic acid	–	–	Married ↑ (<0.001)	–	–	–	–	–	–	–
Iron	–	Female ↑ (0.001)	Married ↑ (0.003)	Expat Arabs ↑ (0.043)	–	No insurance ↑ (0.045)	–	–	–	–
Calcium	–	–	–	Expat Arabs ↑ (0.007)	Employed ↑ (0.049)	No insurance ↑ (0.024)	–	–	Chronic disease Present ↑ (0.039)	–

↑ indicates the subgroup with a higher observed proportion of supplement use within a variable showing a statistically significant overall association. “–” indicates no statistically significant association (*p* ≥ 0.05). Values in parentheses are *p*-values from Pearson’s chi-square test.

*Education was assessed as high school or less versus undergraduate/postgraduate; no significant association was observed with any supplement category or subtype examined.

A *p*-value <0.05 was considered statistically significant.

### 3.5 Patterns and experiences of dietary supplement use

Table 3 summarizes the patterns and experiences of dietary supplement use among ever users. The most commonly reported duration of use was >1 month to <1 year (59.9%). Supplement use was frequent, with 46.1% reporting daily or alternate-day use and 44.4% reporting use one to four times per week. Among participants with available expenditure data, 48.9% spent up to AED 300 per month, and 51.1% spent more than AED 300 per month. Among past users who reported a reason for discontinuation, 66.4% indicated that they had recovered or no longer felt the need to continue supplementation. Most participants perceived dietary supplements to be effective (73.1%), whereas adverse effects (10.2%) and regret regarding supplement use (3.2%) were reported less frequently.

**Table 3:** Patterns and experiences of dietary supplement use among ever users.

Variable	Category	*n*	Valid %
Duration of supplement use (*n* = 284)	Less than 1 month	36	12.7
	>1 month to <1 year	170	59.9
	1–5 years	58	20.4
	More than 5 years	20	7.0
Frequency of supplement use (*n* = 284)	Daily or alternate day	131	46.1
	1–4 times per week	126	44.4
	1–3 times per month	13	4.6
	Occasionally	11	3.9
	Once a month throughout the year	3	1.1
Monthly expenditure (*n* = 221)	Up to AED 300	108	48.9
	More than AED 300	113	51.1
Reason for discontinuation (Past users only, *n* = 110)	Recovered/no longer felt the need to continue	73	66.4
	Costly	16	14.5
	Other reasons	12	10.9
	Allergic reaction	9	8.2
Perceived effectiveness (*n* = 286)	Yes	209	73.1
	Do not know	71	24.8
	No	6	2.1
Adverse effects (*n* = 284)	No	255	89.8
	Yes	29	10.2
Regret using supplements (*n* = 284)	No	275	96.8
	Yes	9	3.2

Among ever users, tablets were the most commonly reported dosage form (71.9%), followed by powders (48.8%). Liquid formulations (18.2%) and chewable or gummy supplements (16.5%) were reported less frequently. As participants could select more than one dosage form, percentages exceeded 100%.

### 3.6 Factors associated with dietary supplement use

Table 4 presents the crude and adjusted associations between participant characteristics and ever use of dietary supplements. In the adjusted model, undergraduate/postgraduate education was associated with substantially higher odds of dietary supplement use compared with high school education or less (AOR 18.32, 95% CI 9.09–36.89; *p* < 0.001). Higher odds of supplement use were also observed among women (AOR 3.13, 95% CI 1.76–5.58; *p* < 0.001), Expat Arabs compared with UAE nationals (AOR 4.58, 95% CI 2.14–9.82; *p* < 0.001), and participants with chronic medical conditions (AOR 2.15, 95% CI 1.27–3.64; *p* = 0.004). Employed participants had lower odds of supplement use than students (AOR 0.28, 95% CI 0.13–0.61; *p* = 0.001). No other variables in the adjusted model were independently associated with dietary supplement use.

**Table 4:** Binary logistic regression analysis of factors associated with ever use of dietary supplements among adults in the UAE.

Variable	Category	COR (95% CI)	Crude *p*-value	AOR (95% CI)	Adjusted p-value
Age group	Young adults, 18–29 years	Reference	–	Reference	–
	Middle-aged adults, 30–49 years	1.11 (0.78–1.59)	0.550	1.77 (0.93–3.39)	0.083
	Older adults, ≥50 years	0.82 (0.42–1.61)	0.562	2.73 (0.82–9.06)	0.102
Age group: overall *p*-value			0.657		0.121
Sex	Male	Reference	–	Reference	–
	Female	2.66 (1.90–3.72)	<0.001	3.13 (1.76–5.58)	<0.001
Nationality	UAE national	Reference	–	Reference	–
	South Asian	0.32 (0.21–0.50)	<0.001	1.00 (0.45–2.22)	0.999
	Expat Arab	5.06 (2.83–9.06)	<0.001	4.58 (2.14–9.82)	<0.001
Nationality: overall *p*-value			<0.001		<0.001
Education	High school or less	Reference	–	Reference	–
	Undergraduate/postgraduate	9.20 (6.34–13.35)	<0.001	18.32 (9.09–36.89)	<0.001
Occupation	Student	Reference	–	Reference	–
	Employed	1.17 (0.84–1.63)	0.349	0.28 (0.13–0.61)	0.001
	Currently not employed	2.65 (0.98–7.18)	0.056	0.22 (0.04–1.15)	0.073
Occupation: overall *p*-value			0.136		0.005
Monthly income	<AED 5,000	Reference	–	Reference	–
	AED 5,000–9,999	2.47 (1.48–4.11)	<0.001	1.46 (0.75–2.84)	0.269
	AED 10,000–19,999	3.39 (1.78–6.48)	<0.001	1.12 (0.45–2.75)	0.809
	≥AED 20,000	2.76 (1.68–4.52)	<0.001	1.55 (0.71–3.34)	0.270
Monthly income: overall *p*-value			<0.001		0.589
Health-insurance coverage	No	Reference	–	Reference	–
	Yes	2.55 (1.64–3.96)	<0.001	0.80 (0.35–1.79)	0.578
Frequency of doctor visits	Once monthly	Reference	–	Reference	–
	Once every 2–6 months	0.17 (0.05–0.57)	0.004	0.18 (0.03–1.03)	0.054
	Once yearly or less	0.09 (0.03–0.31)	<0.001	0.18 (0.03–1.04)	0.056
Doctor-visit frequency: overall *p*-value			<0.001		0.151
Chronic medical condition	No	Reference	–	Reference	–
	Yes	2.56 (1.82–3.60)	<0.001	2.15 (1.27–3.64)	0.004
Prescription-drug use	No	Reference	–	Reference	–
	Yes	1.85 (1.20–2.86)	0.006	1.69 (0.88–3.25)	0.114
Smoking status	Non-smoker	Reference	–	Reference	–
	Current smoker	0.36 (0.26–0.52)	<0.001	0.80 (0.45–1.39)	0.425
	Past smoker	0.48 (0.27–0.83)	0.009	0.77 (0.35–1.69)	0.515
Smoking status: overall *p*-value			<0.001		0.688

COR, crude odds ratio; AOR, adjusted odds ratio; CI, confidence interval.

Outcome variable: Ever use of dietary supplements (current or past use) versus never use.

Reference groups: young adults aged 18–29 years; male; UAE national; high school education or less; student; monthly income <AED 5,000; no health insurance coverage; monthly doctor visits; no chronic medical condition; no prescription drug use; and non-smoker.

Adjusted model: All covariates were entered simultaneously using the Enter method. The adjusted analysis included 539 participants with complete data. No problematic multicollinearity was identified (tolerance: 0.449–0.943; VIF: 1.060–2.228). The model was statistically significant, *χ*^2^(18) = 262.04, *p* < 0.001; Nagelkerke *R*^2^ = 0.515. The Hosmer–Lemeshow goodness-of-fit test was non-significant, *χ*^2^(8) = 8.19, *p* = 0.415, and overall classification accuracy was 80.0%.

A *p*-value <0.05 was considered statistically significant.

### 3.7 Adjusted associations with dietary supplement use

Figure 3 visually presents the magnitude and precision of selected adjusted associations with ever use of dietary supplements. The strongest positive association was observed for undergraduate/postgraduate education, followed by Expat Arab nationality, female sex, and chronic medical conditions, whereas employment was associated with lower odds of supplement use than student status. South Asian nationality and current unemployment were not significantly associated with supplement use after multivariable adjustment.

**Figure 3:**
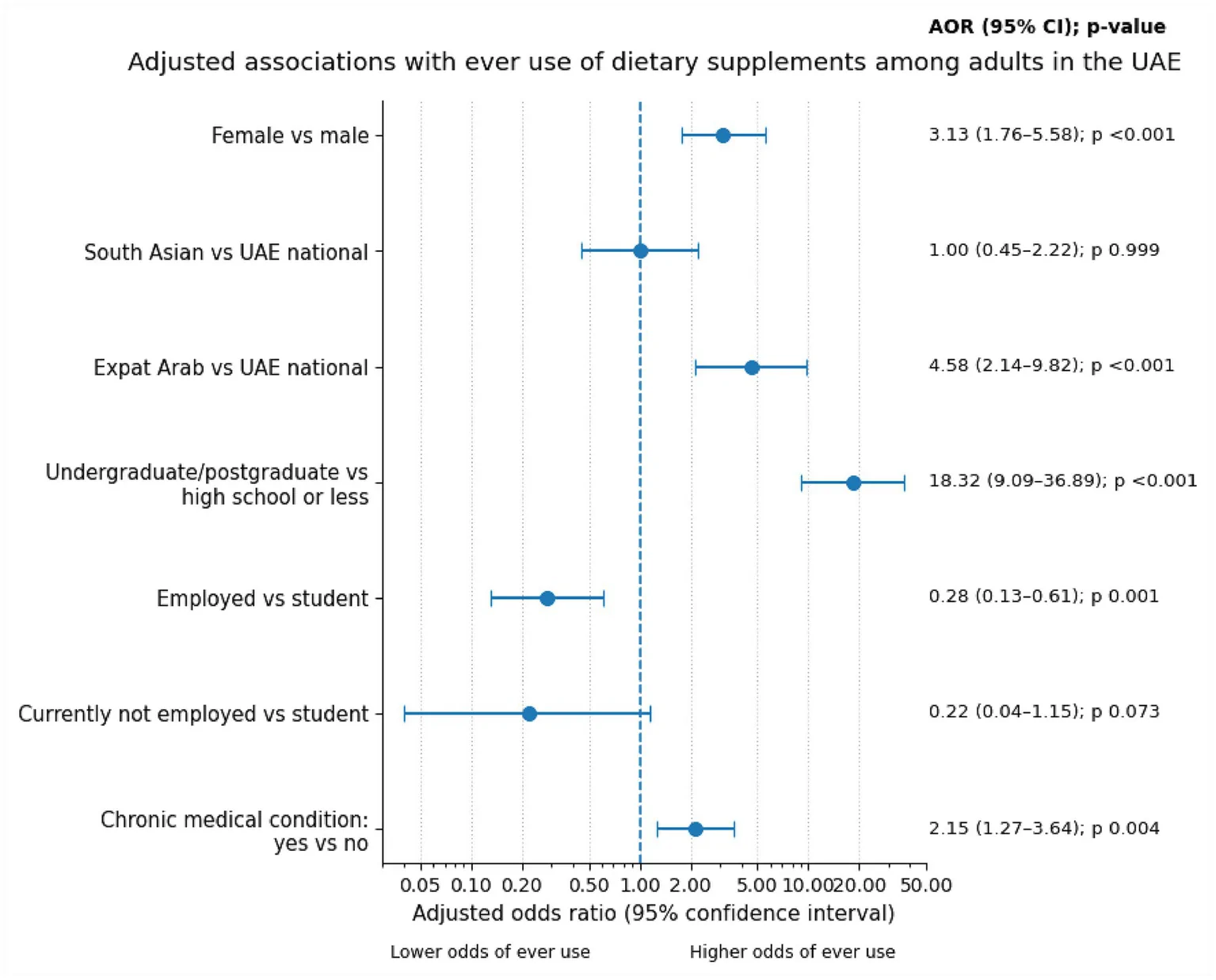
Selected adjusted associations with ever use of dietary supplements among adults in the UAE. Adjusted odds ratios (AORs) and 95% confidence intervals (CIs) are shown for selected participant characteristics from the multivariable binary logistic regression model. The model included age, sex, nationality, education, occupation, monthly income, health insurance coverage, frequency of doctor visits, chronic medical condition, prescription drug use, and smoking status, entered simultaneously using the Enter method. Circles represent AORs, and horizontal lines represent 95% CIs. The vertical dashed line at an odds ratio of 1.00 represents the null value. Complete results for all covariates are presented in Table 4.

### 3.8 Perceptions regarding dietary supplements

Table 5 compares agreement with key perception statements by dietary supplement use status. Ever users showed significantly higher agreement than never users across all four perception statements (all *p* < 0.001). The largest difference was observed for the belief that dietary supplements are necessary for all ages (87.4% vs. 11.8%). Overall, agreement with favorable perceptions regarding dietary supplements was consistently higher among ever users than never users.

**Table 5:** Agreement with key perception statements according to dietary supplement-use status.

Perception statement	Never users (*n* = 313), *n* (%)	Ever users (*n* = 286), *n* (%)	*p*-value
Dietary supplements are necessary for all ages	37 (11.8%)	250 (87.4%)	<0.001
Dietary supplements are generally harmless	84 (26.8%)	233 (81.5%)	<0.001
Regular use of dietary supplements prevents chronic diseases	62 (19.8%)	174 (60.8%)	<0.001
Health personnel should promote the use of dietary supplements	89 (28.5%)	236 (82.5%)	<0.001

Values are presented as *n* (%). Percentages represent the proportion within each dietary supplement-use group who agreed with each perception statement. Group comparisons were performed using Pearson’s chi-square test. One ever user had missing perception data.

A *p*-value <0.05 was considered statistically significant.

### 3.9 Factors associated with dietary supplement perceptions

Table 6 summarizes the adjusted associations with the four dietary supplement perception statements; complete regression models are provided in Supplementary Tables S2A–D. Ever-use of dietary supplements was the only factor consistently associated with all four perceptions. Compared with never users, ever users had substantially higher odds of agreeing that dietary supplements are necessary for all ages, are generally harmless, help prevent chronic diseases, and should be promoted by healthcare personnel (all *p* < 0.001). Other sociodemographic and health-related factors were associated with individual perception statements, but these associations were not consistent across the four models.

**Table 6:** Factors associated with perceptions regarding dietary supplements among adults in the UAE.

Perception statement	Predictor and comparison	COR (95% CI)	Crude *p*-value	AOR (95% CI)	Adjusted *p*-value
Dietary supplements are necessary for all ages	Middle-aged vs. young adults	1.36 (0.96–1.94)	0.088	2.75 (1.09–6.95)	0.033
	Health insurance: Yes vs. No	2.99 (1.91–4.69)	<0.001	2.89 (1.10–7.57)	0.031
	Monthly income AED 5,000–9,999 vs. < AED 5,000	1.98 (1.20–3.28)	0.008	0.35 (0.13–0.90)	0.027
	Undergraduate/postgraduate vs. high school or less	8.98 (6.19–13.02)	<0.001	2.78 (1.14–6.81)	0.024
	Chronic medical condition: Yes vs. No	1.69 (1.21–2.35)	0.002	0.42 (0.18–0.90)	0.025
	Ever supplement user vs. never user	51.80 (31.74–84.53)	<0.001	86.84 (39.66–190.16)	<0.001
Dietary supplements are generally harmless	Older vs. young adults	0.43 (0.21–0.86)	0.017	0.26 (0.09–0.76)	0.013
	Health insurance: Yes vs. No	0.84 (0.55–1.26)	0.393	0.28 (0.13–0.62)	0.002
	Ever supplement user vs. never user	11.99 (8.12–17.69)	<0.001	14.97 (8.49–26.39)	<0.001
Regular supplement use prevents chronic diseases	Expat Arab vs. UAE national	0.62 (0.38–1.00)	0.050	0.41 (0.22–0.80)	0.009
	Employed vs. student	0.62 (0.44–0.86)	0.005	0.37 (0.19–0.73)	0.004
	Currently not employed vs. student	1.07 (0.42–2.73)	0.881	0.18 (0.04–0.94)	0.042
	Monthly income AED 5,000–9,999 vs. < AED 5,000	2.69 (1.62–4.47)	<0.001	2.43 (1.30–4.54)	0.005
	Monthly income AED 10,000–19,999 vs. < AED 5,000	4.26 (2.26–8.04)	<0.001	3.70 (1.53–8.94)	0.004
	Monthly income ≥AED 20,000 vs. < AED 5,000	3.78 (2.30–6.19)	<0.001	4.12 (1.92–8.85)	<0.001
	Prescription-drug use: Yes vs. No	1.90 (1.23–2.92)	0.004	2.44 (1.31–4.53)	0.005
	Ever supplement user vs. never user	6.29 (4.36–9.07)	<0.001	8.36 (4.81–14.53)	<0.001
Health personnel should promote the use of dietary supplements	Ever supplement user vs. never user	11.83 (7.99–17.50)	<0.001	11.13 (6.37–19.44)	<0.001

COR, crude odds ratio; AOR, adjusted odds ratio; CI, confidence interval.

Outcome coding: For each perception statement, agreement was coded as 1 and disagreement as 0.

Adjusted models: Separate multivariable binary logistic regression models were constructed for each perception statement. All models simultaneously included age, sex, nationality, occupation, health insurance coverage, monthly income, education, doctor-visit frequency, chronic medical condition, prescription drug use, smoking status, and dietary supplement use status.

Table presentation: Only predictors with statistically significant adjusted associations (*p* < 0.05) are shown. Complete regression models, including all covariates entered, are provided in Supplementary Tables S2A–D.

A *p*-value <0.05 was considered statistically significant.

## 4 Discussion

Nearly one-half of the participants (47.8%) reported current or previous dietary supplement use, indicating that supplement use is common among adults in the UAE. This prevalence is broadly comparable to previous reports from the UAE and neighboring Gulf countries, although direct comparisons should be made cautiously due to differences in study populations, sampling methods, reference periods, and definitions of supplement use (12, 18, 24). Recent regional studies have similarly reported substantial supplement use among physically active adults in Eastern Saudi Arabia (25) and gym attendees in Jordan (26), although their population-specific sampling limits direct comparison with the broader adult population examined here.

Ever and never users differed across several sociodemographic and health characteristics, particularly sex, education, and chronic medical conditions. Similar associations have been reported in Bahrain, Poland, and Greece (11, 15, 27), while recent Gulf evidence also suggests that educational and occupational characteristics may influence supplement use (25). Awareness was substantially higher among ever users than never users (98.6% vs. 81.0%), consistent with previous reports linking awareness and supplement use (15, 28). However, greater awareness may encourage supplement use, while previous use may itself increase familiarity with these products.

Several other characteristics, including monthly income, health insurance coverage, healthcare utilization, prescription drug use, and smoking status, were associated with supplement use in univariable analyses but were not significant after multivariable adjustment. This attenuation suggests that some crude associations may reflect interrelationships among sociodemographic and health-related characteristics rather than independent effects. Similar variability in determinants has been reported across populations (11, 16), emphasizing the importance of adjusted analyses when identifying independent factors associated with supplement use.

Vitamins (70.6%) and minerals (57.8%) were the most frequently reported supplement categories, followed by protein supplements (47.4%). Similar patterns have been reported in the UAE, Kuwait, and Bahrain, where vitamins and minerals were among the most commonly used supplements (12, 15, 19). Their predominance may partly reflect the broader nutritional context of the UAE, where vitamin D deficiency and other micronutrient concerns have been documented (29). However, nutritional status and clinical indications were not assessed, and it cannot be determined whether supplementation reflected confirmed deficiencies, professional recommendations, or perceived health benefits.

Protein supplements were also commonly reported, consistent with the finding that improving fitness is a leading motivation for supplement use. Qudah et al. (21) similarly reported protein supplement use among UAE university students in association with exercise- and fitness-related practices, while recent evidence from physically active adults in Saudi Arabia highlights fitness, muscle development, and performance-related goals as important motivations (25, 30). However, individual supplement categories were not linked to specific motivations in the present study, and physical activity was not assessed.

Managing nutritional deficiencies (38.0%) and improving fitness (35.3%) were the leading reasons for supplement use, consistent with previous studies that identified health maintenance and the correction of perceived or confirmed nutritional deficiencies as common motivations (14, 28). Healthcare professionals were the most frequently reported source of recommendation (40.1%), although fewer than half of users reported receiving professional advice. Friends (21.8%) and self-initiation (14.4%) also contributed substantially, indicating that many supplement-use decisions occurred outside formal healthcare settings. This combination of professional and informal influences is consistent with previous Gulf and international evidence (12, 14). A recent Saudi study among physically active adults identified social media as a more prominent source of information than healthcare professionals (30), illustrating how information sources may vary across populations and reinforcing the need for accessible, evidence-based guidance.

Associations with participant characteristics varied across individual supplement categories, supporting the value of examining specific products rather than treating supplementation as homogeneous behavior (31). Women more frequently used vitamin, mineral, and iron supplements, while married participants more frequently reported vitamin, mineral, folic acid, and iron supplementation. Protein and performance supplements were more common among men, while age- and health-related associations also differed across products. Such variation may reflect differences in health needs, lifestyle practices, and life-stage nutritional requirements (31). However, pregnancy status, physical activity, clinical indications and laboratory-confirmed deficiencies were not assessed; these findings should therefore be interpreted as demographic associations rather than evidence of specific motivations or clinical needs. Vitamin D supplementation should likewise be considered within the broader UAE context of documented vitamin D deficiency (29), without assuming that supplementation in individual participants reflected confirmed deficiency.

Notably, educational attainment was not associated with any individual supplement category, although it showed the strongest independent association with overall supplement use. This may indicate that education is more closely associated with the general decision to use supplements than with the selection of a particular product, although product choice may also reflect health needs, lifestyle, life stage, and personal preferences that were not examined directly.

Supplement use was generally regular, with more than 90% of ever-users reporting use at least weekly, although most had used supplements for less than 1 year. Thus, supplementation appeared to be incorporated into routine health practices among many users, but regular use should not necessarily be interpreted as long-term persistence (32, 33). Among participants who reported expenditure, 51.1% spent more than AED 300 per month, suggesting that supplementation may be a meaningful out-of-pocket expense for some users; however, expenditure was not associated with the number or type of products purchased (34). Among past users, discontinuation was most commonly attributed to recovery or no longer perceiving a need for supplementation (66.4%), while cost and adverse experiences were less frequently reported (33, 35). Because clinical indications and treatment outcomes were not assessed, perceived recovery should not be interpreted as evidence that the intended health benefit was achieved.

Most users perceived dietary supplements to be effective (73.1%), whereas adverse effects (10.2%) and regret (3.2%) were relatively uncommon. These findings indicate generally favorable self-reported experiences but do not establish clinical efficacy or safety. Previous research has similarly shown favorable perceptions of the benefits and safety of supplements among users (7, 13). Recent UAE evidence concerning non-prescription weight-loss supplements has also highlighted adverse effects and gaps in consumer risk awareness (36), emphasizing the distinction between perceived and established safety.

Educational attainment showed the strongest independent association with dietary supplement use. Participants with undergraduate or postgraduate education had substantially higher odds of ever use than those with high school education or less (AOR 18.32, 95% CI 9.09–36.89). Higher educational attainment has also been associated with supplement use in other populations (11, 16) and recent regional studies (25, 37). Greater access to health and nutrition information or engagement in health-related behaviors may contribute to this association, although these mechanisms were not assessed. The unusually large effect estimate and relatively wide confidence interval warrant cautious interpretation and may reflect the sample’s educational distribution, convenience sampling, or residual confounding.

Female participants had approximately threefold higher adjusted odds of supplement use than male participants (AOR 3.13, 95% CI 1.76–5.58), consistent with previous evidence linking female sex to greater supplement use (11). However, sex-related patterns are not uniform across populations; recent studies among physically active adults in Saudi Arabia and gym attendees in Jordan reported greater use among men (26, 30), potentially reflecting differences in the populations studied and the types and motivations for supplementation.

Expat Arab participants also had higher adjusted odds of supplement use than UAE nationals (AOR 4.58, 95% CI 2.14–9.82). Evidence regarding nationality-specific differences within the UAE remains limited. The association may reflect unmeasured differences in dietary practices, health beliefs, healthcare utilization, or other social and cultural factors; nationality should therefore be regarded as a broad demographic marker rather than a direct explanatory factor.

Participants with chronic medical conditions had approximately twice the odds of supplement use (AOR 2.15, 95% CI 1.27–3.64), consistent with previous reports (33, 35). This may reflect greater healthcare contact or health-related concerns, although the present study did not establish whether supplementation was clinically indicated, professionally recommended, or self-initiated. Employment, in contrast, was associated with lower adjusted odds than student status (AOR 0.28, 95% CI 0.13–0.61), despite no significant crude association. This change after adjustment suggests possible confounding or suppression by other covariates and, given the lack of a consistent corresponding pattern in previous studies, warrants cautious interpretation.

Monthly income, health-insurance coverage, doctor-visit frequency, prescription-drug use, and smoking status were not independently associated with supplement use after adjustment. Their crude associations, therefore, appear to have been attenuated after accounting for other measured characteristics. Overall, the model demonstrated acceptable fit and substantial explanatory capacity, although the associations require confirmation in more representative populations.

Previous supplement use showed the most consistent association with perceptions. Ever users were substantially more likely than never users to agree that dietary supplements are necessary for all ages, are generally harmless, help prevent chronic diseases, and should be promoted by healthcare personnel, consistent with previous studies showing more favorable perceptions among supplement users (13–15). Supplement-use status was the only factor independently associated with all four perception outcomes. The particularly large association with the belief that supplements are necessary for all ages (AOR 86.84) should be interpreted cautiously because of its wide confidence interval and the pronounced separation between ever and never users. Other sociodemographic and health-related characteristics were associated with individual perception outcomes but did not show a consistent pattern across the four models (38), and some estimates were imprecise because of relatively small subgroups.

The strong relationship between supplement use and favorable perceptions cannot establish directionality. Positive beliefs may encourage supplement use, while previous positive experiences may reinforce favorable beliefs. Moreover, favorable perceptions should not be interpreted as evidence that dietary supplements are universally necessary, harmless, or effective in preventing chronic diseases. Positive beliefs may coexist with incomplete knowledge regarding appropriate indications, excessive intake, adverse effects, and supplement–drug interactions (15, 28). Recent UAE evidence among community pharmacists has also identified gaps in supplement-related knowledge despite generally high confidence and frequent engagement with dietary supplements (39). These findings emphasize the importance of individualized, evidence-based counseling that considers dietary intake, clinical need, concurrent medications, and the specific supplement in question, rather than the routine promotion of supplementation.

### 4.1 Strengths and limitations

This study provides an integrated assessment of dietary supplement use among adults in the UAE, encompassing prevalence, product categories, patterns and reasons for use, user experiences, perceptions, and factors associated with both supplement use and supplement-related beliefs. In addition to examining overall supplement use, the study explored sociodemographic and health-related correlates of individual supplement categories and assessed recommendation sources, perceived effectiveness, adverse effects, regret, and supplement-related perceptions, providing a broader understanding of supplement-use behavior than prevalence estimates alone.

Several limitations should be considered. First, the cross-sectional design precludes establishing temporal or causal relationships between participant characteristics, perceptions, and dietary supplement use. Second, convenience sampling and electronic recruitment may have introduced selection bias and limited the generalizability of the findings to the wider UAE adult population. Although recruitment targeted adults residing across the UAE, participants’ emirate of residence was not recorded; therefore, geographic distribution and representativeness across the seven emirates could not be assessed. In addition, the achieved sample size of 600 participants was below the calculated target of 724, which may have reduced statistical precision.

Third, dietary supplement use and related information were self-reported and may be subject to recall and social desirability biases. Fourth, although the questionnaire was informed by previously published instruments, reviewed by subject-matter experts, and pilot-tested, the perception items were not subjected to formal psychometric validation. Fifth, information on product-specific dosage and composition, dietary intake, physical activity, laboratory-confirmed nutritional deficiencies, clinical indications for supplementation, and supplement–drug interactions was not collected, limiting interpretation of some observed associations.

Finally, the analyses of individual supplement categories and perception outcomes involved multiple comparisons and were exploratory; therefore, some statistically significant findings may have occurred by chance and should be interpreted cautiously. Some category-specific and perception-model estimates were also based on relatively small subgroups, resulting in limited precision. Although the multivariable models adjusted for several potential confounders, residual confounding by unmeasured factors cannot be ruled out.

### 4.2 Implications for practice and future research

The widespread use of dietary supplements and the substantial influence of non-professional recommendations highlight the importance of incorporating supplement use history into routine clinical assessment. Healthcare professionals should enquire about supplement use, particularly among individuals with chronic conditions or those taking prescription medications, to identify potentially inappropriate use, excessive intake, and supplement–drug interactions. Evidence-based education should also emphasize that dietary supplements may be appropriate for specific nutritional or clinical indications but should not be regarded as universally necessary, harmless, or effective in preventing chronic diseases. Individualized guidance based on nutritional needs, clinical context, and current evidence may therefore support safer and more appropriate use of supplements.

Future longitudinal studies are needed to clarify the temporal relationship between supplement use and favorable perceptions, while probability-based studies with documented geographic representation across the UAE would strengthen generalizability. Future research should also incorporate detailed information on supplement composition and dosage, clinical indications, dietary intake, physical activity, concurrent medications, and relevant biochemical indicators to better assess the appropriateness, safety, and health consequences of dietary supplement use.

## 5 Conclusion

Dietary supplement use was common among adults in the UAE and varied according to sociodemographic and health-related characteristics. Previous supplement use was also consistently associated with more favorable perceptions regarding dietary supplements. These findings highlight the need for evidence-based nutrition education and individualized professional guidance to support informed and appropriate supplement use. Further longitudinal and population-representative studies are warranted to confirm these associations and to better understand supplement use behaviors and perceptions in the UAE.

## Data Availability

The original contributions presented in the study are included in the article/Supplementary material, further inquiries can be directed to the corresponding author.
