# Supplementary material for: Prevalence, patterns, perceptions, and sociodemographic determinants of dietary supplement use among adults in the United Arab Emirates
Source: Front Nutr. 2026 Oct 13;13:1955988. doi: 10.3389/fnut.2026.1955988 (PMC13710096; doi:10.3389/fnut.2026.1955988)
Supplement: Supplementary file 1 [file Supplementary_file_1.pdf]

Gulf Medical University
Consumer assessment of dietary supplementation: A population-based cross-sectional study in United Arab Emirates

CONSENT FORM

Title: Consumer assessment of dietary supplementation: A population-based cross-sectional study in United Arab Emirates

Investigator: Ali Alkhayat 2023GMD12
Supervisor: Dr Nelofar Sami Khan (Head of the Department of Biomedical Sciences)
Research Site(s): United Arab Emirates

You are invited to participate in the research study titled "Consumer Assessment of Dietary Supplementation."

"This Consent Form gives you information about the study we wish to conduct. You will get to know the benefits you may get if you agree to participate, how we hope to use the information that we will get from this study, and who else will share the information. This will help you to make an informed decision whether to participate or not. You may choose not to participate, or you may withdraw your decision to participate in the study at any time. In either case, you will not lose any benefits to which you are otherwise entitled."

What is the primary aim of this research project:

The aim of this study is to understand the consumer perception and assessment of dietary supplementation among the population of United Arab Emirates.

Why am I being asked to participate in this research study:

The primary objective is to analyse the frequency of dietary supplement usage among the population of United Arab Emirates. The secondary objective is to study the factors influencing the use and population perception on the effectiveness of dietary supplement. Since you fit the inclusion criteria (you are a resident of United Arab Emirates, aged 18 and above), you are invited to participate in this research study.

How long will I be in this study:

Your participation will involve filling out a questionnaire, which is estimated to take approximately 10 minutes to fill it out.

What will I be asked to do:

You will be asked to complete a questionnaire covering socio-demographic information and details about your uses of dietary supplementation. You will also be asked to name the types of dietary supplementation and various other question about your assessment and perception of using them in the provided questionnaire.

What benefit can I expect from participating in the research study:

There will be unfortunately no direct individual benefits. However, by participating, you contribute to valuable insights about consumer perceptions of dietary supplementation, which may enhance understanding in health and wellness.

Confidentiality:

All responses will be anonymous and analysed collectively. Only the research team and the research supervisor, will have access to the data ensuring the absolute confidentiality and anonymity of all individuals who participated in research study.

Feedback:

Results will be published and might be presented in international and local conferences. A copy of the results can be made available upon request.

Consent:

"I have read this Consent Form. All my questions have been answered. I volunteer to take part in this study. I authorize the use and disclosure of my questionnaire's responses to the parties listed in the authorization section of this consent for the purposes described above."

Participant's Signature with Date:

Investigator's Signature and Date:

Section A: Demographic Information

1. Age: ______ years.
2. Gender:
    - Male □
    - Female □
3. Marital Status:
    - Married □
    - Single □
4. Nationality:
    - UAE National □
    - Non-UAE National □ (Specify: ________________)
5. Occupation:
    - Student □
    - Employed □
    - Unemployed □
    - Retired □
6. Health Insurance Coverage:
    - Yes □
    - No □
7. Income:
    - <5000 AED □
    - 5000-<9999 AED □
    - 10000-19999 AED □
    - ≥20000 AED □
8. Education:
    - Less than High School □
    - High School □
    - Graduate □
    - Postgraduate □
9. Weight (kg): ______
10. Height (cm): ______

Section B: Health and Lifestyle

1. Do you have any allergies?
    a. Yes □ (If yes, please specify: ________)
    b. No □
    c. Don't know □
2. How frequently have you visited a doctor in the past 12 months?
    a. At least once a month □
    b. Once in 2-3 months □
    c. Once yearly or less □
3. Have you ever been diagnosed with any serious or chronic medical condition? (You can choose more than one answer)
    a. Respiratory disease: No □, Yes □ (Specify: ________)
    b. Skin disorder: No □, Yes □ (Specify: ________)
    c. Disease of the digestive system: No □, Yes □ (Specify: ________)
    d. Diabetes: No □, Yes □ (Specify: ________)
    e. Cardiovascular disease: No □, Yes □ (Specify: ________)
    f. Cancer: No □, Yes □ (Specify: ________)
    g. Other: No □, Yes □ (Specify: ________)
4. Have you taken prescription drugs in the past month?
    a. Yes □ (Specify: ________)
    b. No □
    c. Don't know □
5. Do you smoke?
    a. Every day □
    b. Occasionally □
    c. In the past □
    d. Never □

Section C: Dietary Supplement Consumption

11. Do you know what health supplements are?
    - Yes □
    - No □
12. Have you ever used health supplements?

- Currently □
- In the past and stopped □
- Never □

13. If you used dietary supplements in the past and stopped, why did you discontinue using any health supplements? (You can choose more than one answer)
    - Allergic reactions □
    - Serious skin disorders □
    - Cost □
    - Others □ (Specify: _________)
14. For how long have you been using/had you used health supplements?
    - Less than a month □
    - More than a month but less than a year □
    - 1-5 years □
    - 5 years □
    - Other □ (Specify: _________)
15. How frequently do/did you use health supplements?
    - Daily or almost daily □
    - 1-4 times a week □
    - 1-3 times a month □
    - Rarer than monthly through the year □
    - Occasionally □
    - Other □ (Specify: _________)
16. Which categories of health supplements do/did you use? (You can choose more than one answer)
    - Vitamins □
    - Minerals □
    - Herbal □
    - Sports nutrition □
    - Energy drink □
    - Dietetic food □
    - Protein supplements
    - Others □ (Specify: ________________)

17. What is the form of the used product(s)? (You can choose more than one answer)
    - Tablet □
    - Powder □
    - Chews/Gummy □
    - Liquid □
    - Other □ (Specify: ________)
18. Which health supplement ingredient(s) do/did you use? (You can choose more than one answer)
    - Vitamin B Complex
    - Vitamin B12
    - Folic Acid
    - Vitamin D
    - Fat Soluble Vitamins
    - Fish Oil
    - Potassium
    - Iron
    - Calcium □
    - Magnesium □
    - Creatine □
    - Fish Oils □
    - Creatine
    - Collagen
    - Glucosamine □
    - Chondroitin □
    - Caffeine □
    - Iron □
    - L-Carnitine □
    - Bee Pollen
    - Melatonin
19. How much approximately are the expenses spent on dietary supplements (Monthly estimate)?

    Less than AED 300

More than AED 300

Section D: Knowledge Assessment:

20. Do you agree or disagree with the current statements:
    - Dietary supplements are necessary for all ages Agree Disagree
    - Dietary supplements are generally harmless Agree Disagree
    - Regular use of supplement prevents chronic diseases Agree Disagree
    - Dietary supplements can prevent cancers Agree Disagree
    - Health personnel should promote use of supplements Agree Disagree
21. How and why did you decide on taking dietary supplements:

----

22. Who advised or recommended you on taking dietary supplements (e.g., Doctor, Pharmacist, Friends, Family members):
    - Doctor □
    - Pharmacist □
    - Friends □
    - Family
    - Social Media
    - No one □
    - Others (specify: -------------------) □
23. Have the dietary supplements achieved the desired effects:
    - Yes □

- No □
- Do not know □
- Do not care □

24. Were you using Dietary supplements to treat a specific disease or disorder such as arthritis, specify the condition you were trying to treat.
25. Were there any adverse effects of using dietary supplements?
    - Yes □
    - No □
    - Do not know □
    - Other (specify: -------------------) □
26. Do you regret using dietary supplements?
    - Yes □
    - No □
27. Why do you or do you not regret using dietary supplements?
