# Supplementary material for: Prevalence, patterns, perceptions, and sociodemographic determinants of dietary supplement use among adults in the United Arab Emirates
Source: Front Nutr. 2026 Oct 13;13:1955988. doi: 10.3389/fnut.2026.1955988 (PMC13710096; doi:10.3389/fnut.2026.1955988)
Supplement: Supplementary file 2 [file Table_1.pdf]

Table S1A. Association between participant characteristics and vitamin supplement use among ever users

Characteristic	Category	Vitamins: No n (%)	Vitamins: Yes n (%)	Total n	p-value
**Age (years)** (n=284)	Young adults (18–29)	59 (70.2)	117 (58.5)	176	0.106
	Middle-aged adults (30–49)	23 (27.4)	69 (34.5)	92	
	Older adults (≥50)	2 (2.4)	14 (7.0)	16	
**Sex** (n=289)	Male	58 (68.2)	77 (37.7)	135	<0.001
	Female	27 (31.8)	127 (62.3)	154	
**Marital status** (n=289)	Single	64 (75.3)	112 (54.9)	176	0.001
	Married	21 (24.7)	92 (45.1)	113	
**Nationality** (n=266)	UAE national	42 (53.8)	116 (61.7)	158	0.281
	South Asian	9 (11.5)	25 (13.3)	34	
	Expat Arab	27 (34.6)	47 (25.0)	74	
**Occupation** (n=289)	Student	35 (41.2)	80 (39.2)	115	0.213
	Employed	49 (57.6)	112 (54.9)	161	
	Currently not employed	1 (1.2)	12 (5.9)	13	
**Health insurance coverage** (n=283)	No	13 (15.3)	21 (10.6)	34	0.266
	Yes	72 (84.7)	177 (89.4)	249	
**Monthly income** (n=280)	<AED 5,000	62 (72.9)	88 (45.1)	150	<0.001
	AED 5,000–9,999	15 (17.6)	30 (15.4)	45	
	AED 10,000–19,999	4 (4.7)	29 (14.9)	33	
	≥AED 20,000	4 (4.7)	48 (24.6)	52	
**Education** (n=289)	High school or less	17 (20.0)	49 (24.0)	66	0.458
	Undergraduate/Postgraduate	68 (80.0)	155 (76.0)	223	
**Chronic medical condition** (n=289)	No	50 (58.8)	99 (48.5)	149	0.111
	Yes	35 (41.2)	105 (51.5)	140	
**Smoking status** (n=288)	Current smoker	42 (49.4)	59 (29.1)	101	0.002
	Past smoker	9 (10.6)	18 (8.9)	27	
	Non-smoker	34 (40.0)	126 (62.1)	160	

Footnote: Data are presented as n (%). Percentages are column percentages. P-values were calculated using Pearson's chi-square test. Variable-specific valid sample sizes may differ because of missing data. A p-value <0.05 was considered statistically significant.

Table S1B. Association between participant characteristics and mineral supplement use among ever users

Characteristic	Category	Minerals: No n (%)	Minerals: Yes n (%)	Total n	p-value
**Age (years)** (n=284)	Young adults (18–29)	86 (71.1)	90 (55.2)	176	0.020
	Middle-aged adults (30–49)	31 (25.6)	61 (37.4)	92	
	Older adults (≥50)	4 (3.3)	12 (7.4)	16	
**Sex** (n=289)	Male	73 (59.8)	62 (37.1)	135	<0.001
	Female	49 (40.2)	105 (62.9)	154	
**Marital status** (n=289)	Single	90 (73.8)	86 (51.5)	176	<0.001
	Married	32 (26.2)	81 (48.5)	113	
**Nationality** (n=266)	UAE national	53 (48.6)	105 (66.9)	158	0.012
	South Asian	18 (16.5)	16 (10.2)	34	
	Expat Arab	38 (34.9)	36 (22.9)	74	
**Occupation** (n=289)	Student	59 (48.4)	56 (33.5)	115	0.024
	Employed	60 (49.2)	101 (60.5)	161	
	Currently not employed	3 (2.5)	10 (6.0)	13	
**Health insurance coverage** (n=283)	No	22 (18.3)	12 (7.4)	34	0.005
	Yes	98 (81.7)	151 (92.6)	249	
**Monthly income** (n=280)	<AED 5,000	79 (67.5)	71 (43.6)	150	<0.001
	AED 5,000–9,999	20 (17.1)	25 (15.3)	45	
	AED 10,000–19,999	9 (7.7)	24 (14.7)	33	
	≥AED 20,000	9 (7.7)	43 (26.4)	52	
**Education** (n=289)	High school or less	33 (27.0)	33 (19.8)	66	0.145
	Undergraduate/Postgraduate	89 (73.0)	134 (80.2)	223	
**Chronic medical condition** (n=289)	No	64 (52.5)	85 (50.9)	149	0.793
	Yes	58 (47.5)	82 (49.1)	140	
**Smoking status** (n=288)	Current smoker	53 (43.4)	48 (28.9)	101	0.035
	Past smoker	11 (9.0)	16 (9.6)	27	
	Non-smoker	58 (47.5)	102 (61.4)	160	

Footnote: Data are presented as n (%). Percentages are column percentages. P-values were calculated using Pearson's chi-square test. Variable-specific valid sample sizes may differ because of missing data. A p-value <0.05 was considered statistically significant.

Table S1C. Association between protein supplement use and participant characteristics among ever users

Characteristic	Category	No Protein Supplement n (%)	Protein Supplement Users n (%)	Total n (%)	P-value
**Age (years)** (n=284)	Young adults (18–29)	99 (67.3)	77 (56.2)	176 (62.0)	0.017
	Middle-aged adults (30–49)	37 (25.2)	55 (40.1)	92 (32.4)	
	Older adults (≥50)	11 (7.5)	5 (3.6)	16 (5.6)	
**Sex** (n=289)	Male	38 (25.0)	97 (70.8)	135 (46.7)	<0.001
	Female	114 (75.0)	40 (29.2)	154 (53.3)	
**Marital status** (n=289)	Single	85 (55.9)	91 (66.4)	176 (60.9)	0.068
	Married	67 (44.1)	46 (33.6)	113 (39.1)	
**Nationality** (n=266)	UAE national	76 (55.1)	82 (64.1)	158 (59.4)	0.321
	South Asian	19 (13.8)	15 (11.7)	34 (12.8)	
	Expat Arab	43 (31.2)	31 (24.2)	74 (27.8)	
**Occupation** (n=289)	Student	71 (46.7)	44 (32.1)	115 (39.8)	0.003
	Employed	71 (46.7)	90 (65.7)	161 (55.7)	
	Currently not employed	10 (6.6)	3 (2.2)	13 (4.5)	
**Health insurance coverage** (n=283)	No	18 (12.2)	16 (11.8)	34 (12.0)	0.901
	Yes	129 (87.8)	120 (88.2)	249 (88.0)	
**Monthly income** (n=280)	<AED 5,000	76 (53.1)	74 (54.0)	150 (53.6)	0.026
	AED 5,000–9,999	15 (10.5)	30 (21.9)	45 (16.1)	
	AED 10,000–19,999	21 (14.7)	12 (8.8)	33 (11.8)	
	≥AED 20,000	31 (21.7)	21 (15.3)	52 (18.6)	
**Education** (n=289)	High school or less	40 (26.3)	26 (19.0)	66 (22.8)	0.138
	Undergraduate/Postgraduate	112 (73.7)	111 (81.0)	223 (77.2)	
**Chronic medical condition** (n=289)	No	74 (48.7)	75 (54.7)	149 (51.6)	0.303
	Yes	78 (51.3)	62 (45.3)	140 (48.4)	
**Smoking status** (n=288)	Current smoker	27 (17.9)	74 (54.0)	101 (35.1)	<0.001
	Past smoker	9 (6.0)	18 (13.1)	27 (9.4)	
	Non-smoker	115 (76.2)	45 (32.8)	160 (55.6)	

Footnote: Data are presented as n (%). Percentages are column percentages. P-values were calculated using Pearson's chi-square test. Variable-specific valid sample sizes may differ because of missing data. A p-value <0.05 was considered statistically significant.

Table S1D. Association between Vitamin D use and participant characteristics among ever users

Characteristic	Category	Did Not Use Vitamin D n (%)	Used Vitamin D n (%)	Total n (%)	P-value
**Age (years)** (n=284)	Young adults (18–29)	58 (49.6)	54 (66.7)	112 (56.6)	0.018
	Middle-aged adults (30–49)	52 (44.4)	20 (24.7)	72 (36.4)	
	Older adults (≥50)	7 (6.0)	7 (8.6)	14 (7.1)	
**Sex** (n=289)	Male	41 (34.7)	31 (37.8)	72 (36.0)	0.658
	Female	77 (65.3)	51 (62.2)	128 (64.0)	
**Marital status** (n=289)	Single	60 (50.8)	46 (56.1)	106 (53.0)	0.464
	Married	58 (49.2)	36 (43.9)	94 (47.0)	
**Nationality** (n=266)	UAE national	82 (71.3)	39 (54.2)	121 (64.7)	0.051
	South Asian	11 (9.6)	13 (18.1)	24 (12.8)	
	Expat Arab	22 (19.1)	20 (27.8)	42 (22.5)	
**Occupation** (n=289)	Student	37 (31.4)	34 (41.5)	71 (35.5)	0.266
	Employed	73 (61.9)	45 (54.9)	118 (59.0)	
	Currently not employed	8 (6.8)	3 (3.7)	11 (5.5)	
**Health insurance coverage** (n=283)	No	7 (6.3)	14 (17.1)	21 (10.8)	0.017
	Yes	105 (93.8)	68 (82.9)	173 (89.2)	
**Monthly income** (n=280)	<AED 5,000	55 (47.8)	37 (47.4)	92 (47.7)	0.148
	AED 5,000–9,999	15 (13.0)	16 (20.5)	31 (16.1)	
	AED 10,000–19,999	12 (10.4)	12 (15.4)	24 (12.4)	
	≥AED 20,000	33 (28.7)	13 (16.7)	46 (23.8)	
**Education** (n=289)	High school or less	22 (18.6)	22 (26.8)	44 (22.0)	0.169
	Undergraduate/Postgraduate	96 (81.4)	60 (73.2)	156 (78.0)	
**Chronic medical condition** (n=289)	No	57 (48.3)	33 (40.2)	90 (45.0)	0.260
	Yes	61 (51.7)	49 (59.8)	110 (55.0)	
**Smoking status** (n=288)	Current smoker	38 (32.2)	18 (22.0)	56 (28.0)	0.248
	Past smoker	11 (9.3)	7 (8.5)	18 (9.0)	
	Non-smoker	69 (58.5)	57 (69.5)	126 (63.0)	

Footnote: Data are presented as n (%). Percentages are column percentages. P-values were calculated using Pearson's chi-square test. Variable-specific valid sample sizes may differ because of missing data. A p-value <0.05 was considered statistically significant.

Table S1E. Association between folic acid use and participant characteristics among ever users

Characteristic	Category	Did Not Use Folic Acid n (%)	Used Folic Acid n (%)	Total n (%)	P-value
**Age (years)** (n=284)	Young adults (18–29)	82 (60.7)	30 (47.6)	112 (56.6)	0.132
	Middle-aged adults (30–49)	46 (34.1)	26 (41.3)	72 (36.4)	
	Older adults (≥50)	7 (5.2)	7 (11.1)	14 (7.1)	
**Sex** (n=289)	Male	54 (40.0)	18 (27.7)	72 (36.0)	0.089
	Female	81 (60.0)	47 (72.3)	128 (64.0)	
**Marital status** (n=289)	Single	84 (62.2)	22 (33.8)	106 (53.0)	<0.001
	Married	51 (37.8)	43 (66.2)	94 (47.0)	

**Nationality** (n=266)	UAE national	81 (64.8)	40 (64.5)	121 (64.7)	0.862
	South Asian	15 (12.0)	9 (14.5)	24 (12.8)	
	Expat Arab	29 (23.2)	13 (21.0)	42 (22.5)	
**Occupation** (n=289)	Student	53 (39.3)	18 (27.7)	71 (35.5)	0.220
	Employed	74 (54.8)	44 (67.7)	118 (59.0)	
	Currently not employed	8 (5.9)	3 (4.6)	11 (5.5)	
**Health insurance coverage** (n=283)	No	14 (10.8)	7 (10.9)	21 (10.8)	0.972
	Yes	116 (89.2)	57 (89.1)	173 (89.2)	
**Monthly income** (n=280)	<AED 5,000	58 (44.6)	34 (54.0)	92 (47.7)	0.505
	AED 5,000–9,999	24 (18.5)	7 (11.1)	31 (16.1)	
	AED 10,000–19,999	17 (13.1)	7 (11.1)	24 (12.4)	
	≥AED 20,000	31 (23.8)	15 (23.8)	46 (23.8)	
**Education** (n=289)	High school or less	31 (23.0)	13 (20.0)	44 (22.0)	0.636
	Undergraduate/Postgraduate	104 (77.0)	52 (80.0)	156 (78.0)	
**Chronic medical condition** (n=289)	No	58 (43.0)	32 (49.2)	90 (45.0)	0.404
	Yes	77 (57.0)	33 (50.8)	110 (55.0)	
**Smoking status** (n=288)	Current smoker	42 (31.1)	14 (21.5)	56 (28.0)	0.252
	Past smoker	10 (7.4)	8 (12.3)	18 (9.0)	
	Non-smoker	83 (61.5)	43 (66.2)	126 (63.0)	

Footnote: Data are presented as n (%). Percentages are column percentages. P-values were calculated using Pearson's chi-square test. Variable-specific valid sample sizes may differ because of missing data. A p-value <0.05 was considered statistically significant.

Table S1F. Association between iron supplement use and participant characteristics among ever users

Characteristic	Category	Did Not Use Iron n (%)	Used Iron n (%)	Total n (%)	P-value
**Age (years)** (n=284)	Young adults (18–29)	42 (53.2)	70 (58.8)	112 (56.6)	0.366
	Middle-aged adults (30–49)	29 (36.7)	43 (36.1)	72 (36.4)	
	Older adults (≥50)	8 (10.1)	6 (5.0)	14 (7.1)	
**Sex** (n=289)	Male	39 (49.4)	33 (27.3)	72 (36.0)	0.001
	Female	40 (50.6)	88 (72.7)	128 (64.0)	
**Marital status** (n=289)	Single	52 (65.8)	54 (44.6)	106 (53.0)	0.003
	Married	27 (34.2)	67 (55.4)	94 (47.0)	
**Nationality** (n=266)	UAE national	52 (73.2)	69 (59.5)	121 (64.7)	0.043
	South Asian	10 (14.1)	14 (12.1)	24 (12.8)	
	Expat Arab	9 (12.7)	33 (28.4)	42 (22.5)	
**Occupation** (n=289)	Student	32 (40.5)	39 (32.2)	71 (35.5)	0.399
	Employed	42 (53.2)	76 (62.8)	118 (59.0)	
	Currently not employed	5 (6.3)	6 (5.0)	11 (5.5)	
**Health insurance coverage** (n=283)	No	4 (5.3)	17 (14.4)	21 (10.8)	0.045
	Yes	72 (94.7)	101 (85.6)	173 (89.2)	
**Monthly income** (n=280)	<AED 5,000	31 (41.9)	61 (51.3)	92 (47.7)	0.651
	AED 5,000–9,999	13 (17.6)	18 (15.1)	31 (16.1)	
	AED 10,000–19,999	10 (13.5)	14 (11.8)	24 (12.4)	
	≥AED 20,000	20 (27.0)	26 (21.8)	46 (23.8)	
**Education** (n=289)	High school or less	20 (25.3)	24 (19.8)	44 (22.0)	0.360
	Undergraduate/Postgraduate	59 (74.7)	97 (80.2)	156 (78.0)	
**Chronic medical condition** (n=289)	No	37 (46.8)	53 (43.8)	90 (45.0)	0.673
	Yes	42 (53.2)	68 (56.2)	110 (55.0)	
**Smoking status** (n=288)	Current smoker	29 (36.7)	27 (22.3)	56 (28.0)	0.080
	Past smoker	7 (8.9)	11 (9.1)	18 (9.0)	
	Non-smoker	43 (54.4)	83 (68.6)	126 (63.0)	

Footnote: Data are presented as n (%). Percentages are column percentages. P-values were calculated using Pearson's chi-square test. Variable-specific valid sample sizes may differ because of missing data. A p-value <0.05 was considered statistically significant.

Table S1G. Association between calcium supplement use and participant characteristics among ever users

Characteristic	Category	Did Not Use Calcium n (%)	Used Calcium n (%)	Total n (%)	P-value
**Age (years)** (n=284)	Young adults (18–29)	70 (57.9)	42 (54.5)	112 (56.6)	0.127
	Middle-aged adults (30–49)	46 (38.0)	26 (33.8)	72 (36.4)	
	Older adults (≥50)	5 (4.1)	9 (11.7)	14 (7.1)	
**Sex** (n=289)	Male	40 (32.8)	32 (41.0)	72 (36.0)	0.236
	Female	82 (67.2)	46 (59.0)	128 (64.0)	
**Marital status** (n=289)	Single	67 (54.9)	39 (50.0)	106 (53.0)	0.497
	Married	55 (45.1)	39 (50.0)	94 (47.0)	
**Nationality** (n=266)	UAE national	82 (73.2)	39 (52.0)	121 (64.7)	0.007
	South Asian	13 (11.6)	11 (14.7)	24 (12.8)	
	Expat Arab	17 (15.2)	25 (33.3)	42 (22.5)	
**Occupation** (n=289)	Student	50 (41.0)	21 (26.9)	71 (35.5)	0.049
	Employed	68 (55.7)	50 (64.1)	118 (59.0)	
	Currently not employed	4 (3.3)	7 (9.0)	11 (5.5)	
**Health insurance coverage** (n=283)	No	8 (6.8)	13 (17.1)	21 (10.8)	0.024
	Yes	110 (93.2)	63 (82.9)	173 (89.2)	
**Monthly income** (n=280)	<AED 5,000	53 (45.7)	39 (50.6)	92 (47.7)	0.653
	AED 5,000–9,999	19 (16.4)	12 (15.6)	31 (16.1)	
	AED 10,000–19,999	13 (11.2)	11 (14.3)	24 (12.4)	
	≥AED 20,000	31 (26.7)	15 (19.5)	46 (23.8)	
**Education** (n=289)	High school or less	29 (23.8)	15 (19.2)	44 (22.0)	0.450

	Undergraduate/Postgraduate	93 (76.2)	63 (80.8)	156 (78.0)	
**Chronic medical condition** (n=289)	No	62 (50.8)	28 (35.9)	90 (45.0)	0.039
	Yes	60 (49.2)	50 (64.1)	110 (55.0)	
**Smoking status** (n=288)	Current smoker	35 (28.7)	21 (26.9)	56 (28.0)	0.603
	Past smoker	9 (7.4)	9 (11.5)	18 (9.0)	
	Non-smoker	78 (63.9)	48 (61.5)	126 (63.0)	

Footnote: Data are presented as n (%). Percentages are column percentages. P-values were calculated using Pearson's chi-square test. Variable-specific valid sample sizes may differ because of missing data. A p-value <0.05 was considered statistically significant.

Table S1H. Association between performance supplement use (energy drinks and sports nutrition) and participant characteristics among ever users

Characteristic	Category	Did Not Use Performance Supplements n (%)	Used Performance Supplements n (%)	Total n (%)	P-value
**Age (years)** (n=284)	Young adults (18–29)	150 (61.7)	26 (63.4)	176 (62.0)	0.818
	Middle-aged adults (30–49)	80 (32.9)	12 (29.3)	92 (32.4)	
	Older adults (≥50)	13 (5.3)	3 (7.3)	16 (5.6)	
**Sex** (n=289)	Male	101 (40.9)	34 (81.0)	135 (46.7)	<0.001
	Female	146 (59.1)	8 (19.0)	154 (53.3)	
**Marital status** (n=289)	Single	144 (58.3)	32 (76.2)	176 (60.9)	0.028
	Married	103 (41.7)	10 (23.8)	113 (39.1)	
**Nationality** (n=266)	UAE national	133 (58.8)	25 (62.5)	158 (59.4)	0.381
	South Asian	27 (11.9)	7 (17.5)	34 (12.8)	
	Expat Arab	66 (29.2)	8 (20.0)	74 (27.8)	
**Occupation** (n=289)	Student	94 (38.1)	21 (50.0)	115 (39.8)	0.323
	Employed	142 (57.5)	19 (45.2)	161 (55.7)	
	Currently not employed	11 (4.5)	2 (4.8)	13 (4.5)	
**Health insurance coverage** (n=283)	No	29 (12.0)	5 (12.2)	34 (12.0)	0.969
	Yes	213 (88.0)	36 (87.8)	249 (88.0)	
**Monthly income** (n=280)	<AED 5,000	132 (55.2)	18 (43.9)	150 (53.6)	0.033
	AED 5,000–9,999	32 (13.4)	13 (31.7)	45 (16.1)	
	AED 10,000–19,999	29 (12.1)	4 (9.8)	33 (11.8)	
	≥AED 20,000	46 (19.2)	6 (14.6)	52 (18.6)	
**Education** (n=289)	High school or less	55 (22.3)	11 (26.2)	66 (22.8)	0.576
	Undergraduate/Postgraduate	192 (77.7)	31 (73.8)	223 (77.2)	
**Chronic medical condition** (n=289)	No	130 (52.6)	19 (45.2)	149 (51.6)	0.375
	Yes	117 (47.4)	23 (54.8)	140 (48.4)	
**Smoking status** (n=288)	Current smoker	82 (33.2)	19 (46.3)	101 (35.1)	0.064
	Past smoker	21 (8.5)	6 (14.6)	27 (9.4)	
	Non-smoker	144 (58.3)	16 (39.0)	160 (55.6)	

Footnote: Data are presented as n (%). Percentages are column percentages. P-values were calculated using Pearson's chi-square test. Variable-specific valid sample sizes may differ because of missing data. A p-value <0.05 was considered statistically significant.
