# Supplementary material for: Prevalence, patterns, perceptions, and sociodemographic determinants of dietary supplement use among adults in the United Arab Emirates
Source: Front Nutr. 2026 Oct 13;13:1955988. doi: 10.3389/fnut.2026.1955988 (PMC13710096; doi:10.3389/fnut.2026.1955988)
Supplement: Supplementary file 3 [file Table_2.pdf]

Table S2A. Factors associated with agreement that "Dietary supplements are necessary for all ages"

Variable	Category	COR (95% CI)	Crude p-value	AOR (95% CI)	Adjusted p-value
**Age group**	Young adults (18–29 years)	Reference	—	Reference	—
	Middle-aged adults (30–49 years)	1.36 (0.96–1.94)	0.088	2.75 (1.09–6.95)	0.033
	Older adults (≥50 years)	0.77 (0.39–1.53)	0.455	1.02 (0.20–5.18)	0.984
**Sex**	Male	Reference	—	Reference	—
	Female	2.26 (1.62–3.15)	<0.001	1.31 (0.62–2.78)	0.477
**Nationality**	UAE national	Reference	—	Reference	—
	South Asian	0.23 (0.15–0.36)	<0.001	0.60 (0.23–1.58)	0.304
	Expat Arab	2.82 (1.67–4.78)	<0.001	0.74 (0.29–1.92)	0.535
**Occupation**	Student	Reference	—	Reference	—
	Employed	1.34 (0.96–1.86)	0.084	0.91 (0.35–2.40)	0.853
	Currently not employed	2.85 (1.05–7.74)	0.040	1.21 (0.12–12.34)	0.872
**Health insurance**	No	Reference	—	Reference	—
	Yes	2.99 (1.91–4.69)	<0.001	2.89 (1.10–7.57)	0.031
**Monthly income**	<AED 5,000	Reference	—	Reference	—
	AED 5,000–9,999	1.98 (1.20–3.28)	0.008	0.35 (0.13–0.90)	0.027
	AED 10,000–19,999	2.77 (1.46–5.23)	0.002	0.33 (0.10–1.15)	0.082
	≥AED 20,000	2.35 (1.44–3.84)	<0.001	0.36 (0.11–1.14)	0.081
**Education**	High school or less	Reference	—	Reference	—
	Undergraduate/Postgraduate	8.98 (6.19–13.02)	<0.001	2.78 (1.14–6.81)	0.024
**Doctor visit frequency**	Once monthly	Reference	—	Reference	—
	Once every 2–6 months	0.37 (0.14–0.97)	0.042	0.69 (0.10–5.01)	0.715
	Once yearly or less	0.22 (0.09–0.56)	0.002	0.50 (0.07–3.68)	0.494
**Chronic medical condition**	No	Reference	—	Reference	—
	Yes	1.69 (1.21–2.35)	0.002	0.42 (0.19–0.90)	0.025
**Prescription drug use**	No	Reference	—	Reference	—
	Yes	1.25 (0.81-1.92)	0.305	0.89 (0.38–2.10)	0.796
**Smoking status**	Non-smoker	Reference	—	Reference	—
	Current smoker	0.43 (0.30–0.61)	<0.001	0.64 (0.30–1.34)	0.237
	Past smoker	0.64 (0.37–1.11)	0.112	0.97 (0.34–2.74)	0.946
**Supplement use status**	Never user	Reference	—	Reference	—
	Ever user	51.80 (31.74–84.53)	<0.001	86.84 (39.66–190.2)	<0.001

Abbreviations: COR, crude odds ratio; AOR, adjusted odds ratio; CI, confidence interval. The outcome was agreement versus disagreement with the respective perception statement, with disagreement as the reference outcome; neutral responses were excluded from the regression analysis. All covariates shown in the table were entered simultaneously using the Enter method. Reference categories are indicated in the table.

Table S2B. Factors associated with agreement that "Dietary supplements are generally harmless"

Variable	Category	COR (95% CI)	Crude p-value	AOR (95% CI)	Adjusted p-value
**Age group**	Young adults (18–29 years)	Reference	—	Reference	—
	Middle-aged adults (30–49 years)	0.94 (0.66–1.34)	0.742	0.62 (0.34–1.14)	0.125
	Older adults (≥50 years)	0.43 (0.21–0.86)	0.017	0.26 (0.09–0.76)	0.013
**Sex**	Male	Reference	—	Reference	—
	Female	1.51 (1.09–2.10)	0.014	0.66 (0.38–1.15)	0.144
**Nationality**	UAE national	Reference	—	Reference	—
	South Asian	0.76 (0.52–1.12)	0.171	1.26 (0.57–2.79)	0.564
	Expat Arab	2.79 (1.66–4.70)	<0.001	0.97 (0.46–2.04)	0.940
**Occupation**	Student	Reference	—	Reference	—
	Employed	1.20 (0.87–1.67)	0.271	0.91 (0.44–1.88)	0.799
	Currently not employed	1.09 (0.43–2.78)	0.851	0.64 (0.13–3.23)	0.590
**Health insurance**	No	Reference	—	Reference	—
	Yes	0.84 (0.55–1.26)	0.393	0.28 (0.13–0.62)	0.002
**Monthly income**	<AED 5,000	Reference	—	Reference	—
	AED 5,000–9,999	1.60 (0.96–2.66)	0.069	1.14 (0.56–2.32)	0.714
	AED 10,000–19,999	2.00 (1.06–3.77)	0.033	1.18 (0.46–3.00)	0.736
	≥AED 20,000	1.26 (0.78–2.03)	0.353	0.70 (0.32–1.53)	0.377
**Education**	High school or less	Reference	—	Reference	—
	Undergraduate/Postgraduate	3.30 (2.36–4.62)	<0.001	1.86 (0.89–3.87)	0.097
**Doctor visit frequency**	Once monthly	Reference	—	Reference	—
	Once every 2–6 months	0.36 (0.13–0.99)	0.048	1.50 (0.38–5.91)	0.561
	Once yearly or less	0.21 (0.08–0.57)	0.002	0.87 (0.22–3.40)	0.842
**Chronic medical condition**	No	Reference	—	Reference	—
	Yes	1.68 (1.20–2.36)	0.002	0.81 (0.49–1.35)	0.421
**Prescription drug use**	No	Reference	—	Reference	—
	Yes	1.77 (1.14–2.76)	0.012	1.44 (0.76–2.71)	0.263
**Smoking status**	Non-smoker	Reference	—	Reference	—
	Current smoker	0.48 (0.34–0.68)	<0.001	0.76 (0.44–1.32)	0.332
	Past smoker	0.50 (0.29–0.87)	0.015	0.74 (0.35–1.56)	0.425
**Supplement use status**	Never user	Reference	—	Reference	—
	Ever user	11.99 (8.12–17.69)	<0.001	14.97 (8.49–26.39)	<0.001

Abbreviations: COR, crude odds ratio; AOR, adjusted odds ratio; CI, confidence interval. The outcome was agreement versus disagreement with the respective perception statement, with disagreement as the reference outcome; neutral responses were excluded from the regression analysis. All covariates shown in the table were entered simultaneously using the Enter method. Reference categories are indicated in the table.

Table S2C. Factors associated with agreement that regular use of dietary supplements prevents chronic diseases

Variable	Category	COR (95% CI)	Crude p-value	AOR (95% CI)	Adjusted p-value
**Age group**	Young adults (18–29 years)	Reference	—	Reference	—
	Middle-aged adults (30–49 years)	0.93 (0.65–1.33)	0.685	1.39 (0.74–2.64)	0.308
	Older adults (≥50 years)	0.78 (0.38–1.56)	0.476	1.83 (0.53–6.30)	0.337
**Sex**	Male	Reference	—	Reference	—
	Female	2.61 (1.86–3.66)	<0.001	1.51 (0.89–2.57)	0.126
**Nationality**	UAE national	Reference	—	Reference	—
	South Asian	0.26 (0.16–0.41)	<0.001	0.75 (0.36–1.55)	0.433
	Expat Arab	0.62 (0.38–1.00)	0.050	0.41 (0.22–0.80)	0.009
**Occupation**	Student	Reference	—	Reference	—
	Employed	0.62 (0.44–0.86)	0.005	0.37 (0.19–0.73)	0.004
	Currently not employed	1.07 (0.42–2.73)	0.881	0.18 (0.04–0.94)	0.042
**Health insurance**	No	Reference	—	Reference	—
	Yes	3.68 (2.20–6.16)	<0.001	1.68 (0.77–3.66)	0.189
**Monthly income**	<AED 5,000	Reference	—	Reference	—
	AED 5,000–9,999	2.69 (1.62–4.47)	<0.001	2.43 (1.30–4.54)	0.005
	AED 10,000–19,999	4.26 (2.26–8.04)	<0.001	3.70 (1.53–8.94)	0.004
	≥AED 20,000	3.78 (2.30–6.19)	<0.001	4.12 (1.92–8.85)	<0.001
**Education**	High school or less	Reference	—	Reference	—
	Undergraduate/Postgraduate	2.14 (1.53–3.00)	<0.001	0.68 (0.34–1.36)	0.276
**Doctor visit frequency**	Once monthly	Reference	—	Reference	—
	Once every 2–6 months	0.53 (0.23–1.25)	0.146	0.83 (0.26–2.62)	0.750
	Once yearly or less	0.25 (0.11–0.58)	0.001	0.54 (0.17–1.71)	0.293
**Chronic medical condition**	No	Reference	—	Reference	—
	Yes	1.07 (0.76–1.50)	0.711	0.69 (0.41–1.14)	0.146
**Prescription drug use**	No	Reference	—	Reference	—
	Yes	1.90 (1.23–2.92)	0.004	2.44 (1.31–4.53)	0.005
**Smoking status**	Non-smoker	Reference	—	Reference	—
	Current smoker	0.56 (0.39–0.79)	0.001	1.10 (0.64–1.89)	0.742
	Past smoker	0.60 (0.34–1.07)	0.084	0.87 (0.39–1.93)	0.729
**Supplement use status**	Never user	Reference	—	Reference	—
	Ever user	6.29 (4.36–9.07)	<0.001	8.36 (4.81–14.53)	<0.001

Abbreviations: COR, crude odds ratio; AOR, adjusted odds ratio; CI, confidence interval. The outcome was agreement versus disagreement with the respective perception statement, with disagreement as the reference outcome; neutral responses were excluded from the regression analysis. All covariates shown in the table were entered simultaneously using the Enter method. Reference categories are indicated in the table.

Table S2D. Factors associated with agreement that health personnel (doctors) should promote the use of dietary supplements

Variable	Category	COR (95% CI)	Crude p-value	AOR (95% CI)	Adjusted p-value
**Age group**	Young adults (18–29 years)	Reference	–	Reference	–
	Middle-aged adults (30–49 years)	0.67 (0.47–0.95)	0.026	0.53 (0.28–1.02)	0.058
	Older adults (≥50 years)	0.46 (0.23–0.91)	0.026	0.58 (0.19–1.84)	0.357
**Sex**	Male	Reference	–	Reference	–
	Female	2.52 (1.79–3.54)	<0.001	1.00 (0.57–1.73)	0.987
**Nationality**	UAE national	Reference	–	Reference	–
	South Asian	0.28 (0.19–0.43)	<0.001	0.69 (0.33–1.45)	0.332
	Expat Arab	2.41 (1.40–4.15)	0.001	1.37 (0.63–2.96)	0.429
**Occupation**	Student	Reference	–	Reference	–
	Employed	0.82 (0.59–1.14)	0.228	1.01 (0.49–2.09)	0.977
	Currently not employed	1.04 (0.40–2.67)	0.941	0.51 (0.10–2.53)	0.407
**Health insurance**	No	Reference	–	Reference	–
	Yes	2.72 (1.77–4.17)	<0.001	1.62 (0.76–3.45)	0.215
**Monthly income**	<AED 5,000	Reference	–	Reference	–
	AED 5,000–9,999	2.36 (1.39–4.01)	0.002	1.54 (0.77–3.10)	0.227
	AED 10,000–19,999	2.51 (1.30–4.84)	0.006	0.86 (0.33–2.25)	0.753
	≥AED 20,000	1.66 (1.02–2.71)	0.041	1.02 (0.46–2.28)	0.960
**Education**	High school or less	Reference	–	Reference	–
	Undergraduate/Postgraduate	3.44 (2.45–4.83)	<0.001	1.04 (0.51–2.13)	0.912
**Doctor visit frequency**	Once monthly	Reference	–	Reference	–
	Once every 2–6 months	0.56 (0.20–1.56)	0.269	1.66 (0.39–6.98)	0.492
	Once yearly or less	0.19 (0.07–0.52)	0.001	0.71 (0.17–2.95)	0.635
**Chronic medical condition**	No	Reference	–	Reference	–
	Yes	1.90 (1.35–2.66)	<0.001	1.19 (0.70–2.01)	0.529
**Prescription drug use**	No	Reference	–	Reference	–
	Yes	1.64 (1.05–2.55)	0.029	1.85 (0.95–3.62)	0.073
**Smoking status**	Non-smoker	Reference	–	Reference	–
	Current smoker	0.42 (0.30–0.60)	<0.001	0.82 (0.47–1.42)	0.477
	Past smoker	0.44 (0.25–0.76)	0.004	0.54 (0.25–1.19)	0.126
**Supplement use status**	Never user	Reference	–	Reference	–
	Ever user	11.83 (7.99–17.50)	<0.001	11.13 (6.37–19.44)	<0.001

Abbreviations: COR, crude odds ratio; AOR, adjusted odds ratio; CI, confidence interval. The outcome was agreement versus disagreement with the respective perception statement, with disagreement as the reference outcome; neutral responses were excluded from the regression analysis. All covariates shown in the table were entered simultaneously using the Enter method. Reference categories are indicated in the table.
